# Comparative analysis of left atrial appendage closure efficacy and outcomes by CHA_2_DS_2_-VASc score group in patients with non-valvular atrial fibrillation

**DOI:** 10.3389/fcvm.2022.905728

**Published:** 2022-07-22

**Authors:** Mingzhong Zhao, Mengxi Zhao, Cody R. Hou, Felix Post, Nora Herold, Jens Walsleben, Qingru Yuan, Zhaohui Meng, Jiangtao Yu

**Affiliations:** ^1^Heart Center, Zhengzhou Ninth People's Hospital, Zhengzhou, China; ^2^Department of Cardiology, Helmut-G.-Walther-Klinikum, Lichtenfels, Germany; ^3^Department of Neurology, Beijing Tiantan Hospital, Capital Medical University, Beijing, China; ^4^Cardiovascular Division, Department of Medicine, University of Minnesota Medical School, Minneapolis, MN, United States; ^5^Clinic for General Internal Medicine and Cardiology, Catholic Medical Center Koblenz-Montabaur, Koblenz, Germany; ^6^Department of Cardiology, Kunming Medical University, Kunming, China

**Keywords:** atrial fibrillation, CHA_2_DS_2_-VASc score, left atrial appendage closure, outcomes, stroke, major bleeding

## Abstract

**Background:**

Higher CHA_2_DS_2_-VASc score is associated with an increased risk of adverse cardio-cerebrovascular events in patients with non-valvular atrial fibrillation (NVAF), regardless of oral anticoagulation (OAC) status. However, whether this association still exists in patients undergoing left atrial appendage closure (LAAC) is unknown. We evaluated the impact of CHA_2_DS_2_-VASc score on LAAC efficacy and outcomes.

**Methods:**

A total of 401 consecutive patients undergoing LAAC were included and divided into 3 groups based on CHA_2_DS_2_-VASc score (0–2, 3–4, and ≥5). Baseline characteristics, periprocedural complications, and long-term outcomes were collected and compared across all groups.

**Results:**

There were no significant differences in implantation success, periprocedural complications, and long-term outcomes across all score groups. Kaplan-Meier estimation showed that the cumulative ratio of freedom from all-cause mortality (*P* = 0.146), cardiovascular mortality (*P* = 0.519), and non-cardiovascular mortality (*P* = 0.168) did not differ significantly by CHA_2_DS_2_-VASc score group. LAAC decreased the risks of thromboembolism and major bleeding, resulting in a relative risk reduction (RRR) of 82.4% (*P* < 0.001) and 66.7% (*P* < 0.001) compared with expected risks in the overall cohort, respectively. Subgroup analysis indicated that observed risks of thromboembolism and major bleeding were significantly lower than the expected risks in score 3–4 and score ≥5 groups, respectively. The level of RRR increased with CHA_2_DS_2_-VASc score (*P* < 0.001 for trend) for thromboembolism but not for major bleeding (*P* = 0.2729 for trend).

**Conclusion:**

Patients with higher CHA_2_DS_2_-VASc score did not experience worse outcomes, which may be partly attributed to more benefits provided by LAAC intervention in such patients compared to those with a low score.

## Introduction

Atrial fibrillation (AF) has been considered a cardiovascular epidemic and is associated with an increased risk of complications such as thromboembolic stroke, heart failure, and death (1, 2). Oral anticoagulation (OAC) therapy is one of the most effective strategies for preventing stroke and reducing the incidence of complications in these patients. Previous studies showed that both traditional warfarin and direct oral anticoagulants (DOACs) decrease the risk of stroke or systemic embolic events (3), where DOACs demonstrated a favorable risk-benefit profile in risk reduction of hemorrhagic stroke, intracranial hemorrhage, and all-cause mortality, but an increased risk of gastrointestinal bleeding compared with warfarin (4). However, OACs face several limitations in their application, especially in patients at higher risk of bleeding and in patients who are not optimal candidates for long-term OAC because of OAC contraindications or non-compliance.

Intracardiac thrombus arising from the left atrial appendage (LAA) is the main cause of thromboembolic stroke in patients with non-valvular AF (NVAF) (5). Percutaneous LAA closure (LAAC) by an occluder is a non-pharmacologic approach to stroke prevention in NVAF patients. The long-term outcomes of randomized clinical trials showed that the efficacy of LAAC with the WATCHMAN device was comparable to warfarin in preventing stroke, with additional reductions in the risk of major bleeding and mortality (6). Furthermore, in patients with a high risk of stroke and bleeding, LAAC was still non-inferior to DOACs in preventing stroke and decreasing bleeding risks (7).

The CHA_2_DS_2_-VASc score has been proved to be significantly associated with the risk of cardiogenic embolism in patients with AF and is commonly used to stratify the risk of future thromboembolism or adverse outcomes in current clinical practice guidelines (8–10). Recent studies demonstrated that the risk of major adverse cardiovascular and cerebrovascular events or mortality in AF patients taking OACs still increased sequentially with increasing CHA_2_DS_2_-VASc score (9, 11). However, data on the relationship between CHA_2_DS_2_-VASc score and clinical outcomes in patients treated with percutaneous LAAC are limited. We investigated the association between CHA_2_DS_2_-VASc score groups and LAAC efficacy and outcomes.

## Methods

### Subjects

This is a retrospective cohort study. Patients with NVAF who consecutively underwent LAAC with the WATCHMAN device (Boston Scientific, Marlborough, MA, USA) at Helmut-G.-Walther Klinikum, Lichtenfels, Germany, from February 2012 to June 2018 and the WATCHMAN device or LAmbre^TM^ occluder (Lifetech Scientific Corp., Shenzhen, China) at Zhengzhou Ninth People's Hospital, Zhengzhou, China from October 2016 throughout September 2021 were enrolled. The inclusion criteria for LAAC were as follows: (1) patients with known NVAF or whose NVAF was newly diagnosed during hospitalization, (2) those with a high risk of stroke or systemic embolism, (3) those with a high risk of major bleeding or contraindication for long-term anticoagulation therapy, or (4) those who were reluctant to take oral anticoagulation drugs. Patients with malignancy or multiple organ failure with a life expectancy of <1 year or patients with an intracardiac thrombus in the left atria/left atrial appendage detected by imaging were excluded. Written informed consent for LAAC was obtained from all participants. The study was carried out based on the Declaration of Helsinki and approved by the Ethics Committee of Helmut-G.-Walther Klinikum, Lichtenfels, Germany and Zhengzhou Ninth People's Hospital, Zhengzhou, China. Patients were divided into three groups based on CHA_2_DS_2_-VASc score, namely, 0–2, 3–4, and ≥5. Data on baseline characteristics, periprocedural complications within 7 days, and long-term outcomes were collected and analyzed.

### Procedure

The detailed protocol of LAAC with the WATCHMAN device has been described previously (12). LAAC was performed under general anesthesia. Intra-procedural transesophageal echocardiography (TEE) and fluoroscopy were used to guide device implantation. After septum puncture, a WATCHMAN occluder was implanted in the left atrial appendage according to the device's directions for use. Implantation of LAmbre devices was generally similar to that of the WATCHMAN device. The occluder was released after the implantation met criteria, such as position, size, sealing effect, and stability. Implantation success was defined as an adequate closure of the LAA without residual peri-device leaks >5 mm in width, absence of device-related thrombus (DRT), and stable position of the device. Patients were hospitalized for 24–48 h, and those without significant procedure-related complications were then discharged.

### Post-procedural antithrombotic treatments

To allow time for device endothelialization post-procedure, patients were prescribed antithrombotic drugs. The postprocedural antithrombotic regimen was at the discretion of the physician based on patients' clinical characteristics.

### Follow-up

Patients were followed through with TEE evaluation and clinical visits. TEE follow-up was performed at approximately 45 days and 6 months after device implantation. Clinical follow-up was scheduled at 45 days and 6 months after the procedure as well as at the end of this study.

### Outcomes

The primary outcomes were the success rate of device implantation and major adverse events during long-term follow-up. The secondary outcomes were severe periprocedural complications within 7 days. Thromboembolism (ischemic stroke/transient ischemic attack [TIA]/systemic embolism), major bleeding (intracranial hemorrhage/gastrointestinal bleeding/other major bleeding), DRT, mortality (cardiovascular mortality/non-cardiovascular mortality), and combined efficacy endpoints (thromboembolism/mortality) were defined as major adverse events. Severe periprocedural complications included ischemic stroke, TIA, systemic embolism, major bleeding, pericardial effusion/cardiac tamponade, severe vascular complication, and device-related death.

### Statistical analysis

We used SPSS version 26.0 (SPSS Inc., Chicago, Illinois) for data analysis. Continuous variables are presented as means with standard deviation. Categorical variables are expressed as counts and proportions in percentage. The trends across the multiple groups were evaluated *via* the Cochran-Armitage test for categorical variables, the exact Cochran-Armitage test for categorical variables with rare events, or the Jonckheere-Terpstra test for metrical variables. The difference between two groups was compared with chi-square (χ^2^) tests for categorical variables, Mann-Whitney-Wilcoxon tests for metrical variables, or Student's *t*-tests for continuous variables. The mortality risk at long-term follow-up after LAAC was assessed by Kaplan-Meier curve analysis. The survival curves show cumulative ratios of freedom from all-cause mortality, cardiovascular mortality, and non-cardiovascular mortality. A log-rank test was used to compare the differences in mortality risk among groups.

The estimated annual rates of thromboembolism and major bleeding were calculated based on patient CHA_2_DS_2_-VASc and HAS-BLED scores, respectively (13, 14). The observed annual rates of thromboembolism and major bleeding were calculated as events per 100 patient-years, which were calculated as the total number of patients in whom new thromboembolism or major bleeding events developed during follow-up in a group divided by the total followed patient-years and then multiplied by 100. The efficacy of LAAC on thromboembolic or major bleeding risks was analyzed by comparing the estimated risk of thromboembolism or major bleeding events and observed risk. A chi-square test with relative risk (RR) and its 95% confidence interval (CI) was used to assess the differences between estimated and observed risks in one group. The number needed to treat (NNT) to prevent one event in thromboembolism or major bleeding by LAAC intervention was calculated in the overall cohort, score 0–2, score 3–4, and score ≥5 groups using the formula NNT = 1 / (predicted annual rate of event—observed annual rate of event).

The relative risk reduction (RRR) in thromboembolic or major bleeding events was calculated using the formula RRR = (estimated annual rate—observed annual rate)/estimated annual rate. To evaluate the differences regarding the impact of LAAC on thromboembolic and major bleeding outcomes in patients by CHA_2_DS_2_-VASc score group, the comparisons of RRR in thromboembolism or major bleeding were performed and analyzed by a chi-square test among groups, respectively. A *P*-value < 0.05 was considered statistically significant.

## Results

### Baseline characteristics

A total of 411 consecutive patients with NVAF were scheduled to undergo LAAC. LAAC was performed successfully in 401 patients (mean age 74.9 ± 8.0 years; 65.8% male) who were subsequently enrolled in this study. Among the other 10 patients, LAA morphology was not suitable for WATCHMAN in 6 cases, repeated DRT occurred in 1 case, and cardiac tamponade occurred in 2 cases at Helmut-G.-Walther Klinikum, Lichtenfels, Germany; severe iliac vein stenosis after pelvic surgery presented in 1 case at Zhengzhou Ninth People's Hospital, Zhengzhou, China. Among the overall cohort, 75 cases had scores 0–2, 188 cases had scores 3–4, and 138 cases had scores ≥5 according to CHA_2_DS_2_-VASc score. Out of the 75 patients with scores 0–2, 2 cases had 0 score whose indication for LAAC was gastrointestinal bleeding and OAC incompliance for each 1 case, and 10 cases had 1 score whose indication for LAAC was gastrointestinal bleeding in 5 cases, OAC incompliance in 2 cases, anemia in 1 case, and refusal of OAC in 2 cases. The total implantation success was 97.6%. There was no statistical difference for device implant success among groups (score 0–2: 97.3%; score 3–4: 97.8%; score ≥5: 97.5%; *P* = 0.965 for trend). The baseline characteristics of patients are presented in Table 1. Patients in the score ≥5 group were oldest (*P* < 0.001); most likely to be female; and have hypertension, coronary heart disease (CHD), diabetes mellitus, congestive heart failure (CHF), previous stroke, previous major bleeding, and chronic kidney disease (CKD) (each *P* < 0.01 for trend), while the proportion of patients with abnormal liver function was lowest in this group (*P* = 0.006 for trend). HAS-BLED score increased with increasing CHA_2_DS_2_-VASc scores (*P* < 0.001 for trend). However, the type of NVAF was comparable among all groups (Table 1).

**Table 1:** Patient baseline characteristics.

**Variables**	**Overall cohort**	**Score 0–2**	**Score 3–4**	**Score** ≥**5**	***P*** **-value for trend**
	***n*** = **401**	***n*** = **75**	***n*** = **188**	***n*** = **138**	
Age, years (mean ± SD)	74.9 ± 8.0	67.6 ± 8.7	75.0 ± 7.1	78.6 ± 6.0	<0.001
≥75 years, *n* (%)	235(58.6)	14 (18.7)	105 (55.9)	116 (84.1)	<0.001
Male, *n* (%)	264 (65.8)	64 (85.3)	130 (69.2)	70 (50.7)	<0.001
Hypertension, *n* (%)	317(79.1)	42 (56.0)	153 (81.4)	122 (88.4)	<0.001
CHD, *n* (%)	203(50.6)	31 (41.3)	82 (43.6)	90 (65.2)	<0.001
Diabetes mellitus, *n* (%)	111(27.7)	8 (10.7)	37 (19.7)	66 (47.8)	<0.001
CHF^▴^, *n* (%)	85(21.2)	2 (2.7)	30 (16.0)	53 (38.4)	<0.001
Previous stroke, *n* (%)	91(22.7)	5 (6.7)	23 (12.2)	63 (45.7)	<0.001
Previous major bleeding, *n* (%)	134(33.4)	19 (25.3)	57 (30.3)	58 (42.0)	0.008
CKD^♦^, *n* (%)	171(42.6)	17 (22.7)	84 (44.7)	70 (50.7)	<0.001
Abnormal liver function^⋆^, *n* (%)	49(12.2)	12 (16.0)	26 (13.8)	11 (8.0)	0.006
HAS-BLED score (mean ± SD)	3.5 ± 1.1	2.6 ± 1.0	3.5 ± 0.9	4.0 ± 0.9	<0.001
AF, paroxysmal, *n* (%)	142 (35.4)	29 (38.7)	64 (34.0)	49 (35.5)	0.735
AF, persistent or permanent, *n* (%)	259(64.6)	46 (61.3)	124 (66.0)	89 (64.5)	0.735

*Continuous data are reported as means and standard deviation. Categorical variables are expressed as frequencies (n) and percentages (%). ^▴^defined as the presence of left ventricular ejection fraction (LVEF) <40% or symptomatic heart failure; ^♦^defined as an estimated glomerular filtration rate (eGFR) <60 ml/min/1.73 m^2^; ^⋆^ defined as prior liver disease or presence of elevated liver enzymes (alanine aminotransferase/aspartate aminotransferase ≥ 2 × upper limit of normal) at admission. CHD, coronary heart disease; CHF, congestive heart failure; CKD, chronic kidney disease; AF, atrial fibrillation*.

### Periprocedural complications

The total incidence of periprocedural complications was 3.2%, with 1.3%, 3.2%, and 4.3% in score 0–2, score 3–4, and score ≥5 groups, respectively (Table 2). No device-related death was observed in the overall cohort. There were no significant trends in the incidence of complications such as stroke, major bleeding, pericardial effusion/cardiac tamponade, and severe vascular complication among the three groups.

**Table 2:** Periprocedural complications within 7 days.

**Variables**	**Overall cohort**	**Score 0-2**	**Score 3–4**	**Score** ≥**5**	***P*** **–value for trend**
	***n*** = **401**	***n*** = **75**	***n*** = **188**	***n*** = **138**	
Stroke, *n* (%)	1 (0.3)	0 [0]	1 (0.5)	0 (0)	0.825
TIA, *n* (%)	0 (0)	0 (0)	0 (0)	0 (0)	—
Other systemic embolism, *n* (%)	0 (0)	0 (0)	0 (0)	0 (0)	—
Major bleeding, *n* (%)	2 (0.5)	0 (0)	1 (0.5)	1 (0.7)	0.495
Pericardial effusion/cardiac tamponade, *n* (%)	4 (1.0)	0 (0)	1 (0.5)	3 (2.2)	0.094
Severe vascular complication, *n* (%)	6 (1.5)	1 (1.3)	3 (1.6)	2 (1.5)	0.974
Device-related death, *n* (%)	0 (0)	0 (0)	0 (0)	0 (0)	—
Total, *n* (%)	13 (3.2)	1 (1.3)	6 (3.2)	6 (4.3)	0.241

*Categorical variables are expressed as frequencies (n) and percentages (%). TIA, transient ischemic attack*.

### Antithrombotic regimen post-LAAC

Variation in antithrombotic regimens occurred mainly at the first 45 days after the procedure. During their hospital stay post-LAAC, patients were prescribed warfarin, aspirin plus warfarin, aspirin plus low-molecular-weight heparin (LMWH), a combination of aspirin and DOACs, aspirin plus clopidogrel, or no antithrombotic medications; most patients (58.9 %) were treated with aspirin plus LMWH (Table 3). No significant trends were found for these regimens among the three groups for the duration of hospital stay post-LAAC except for aspirin plus LMWH which was more often prescribed in the score 0–2 group, and aspirin plus DOACs with the highest proportion in score ≥5 group. For the period from discharge to 45 days post-LAAC, all the antithrombotic regimens were continued except for subcutaneous injection of LMWH, which was switched to oral antithrombotics. The antithrombotic strategies for this period were also comparable among groups (Table 3). At the 45-day visit post-procedure, if TEE imaging identified adequate closure of the LAA (no residual peri-device jet >5 mm) without DRT, the antithrombotic regimen was switched to aspirin plus clopidogrel until 6 months post-procedure. Aspirin alone was administered indefinitely if TEE indicated adequate closure without DRT at the 6-month visit. If TEE showed inadequate closure or a presence of DRT, OACs were restarted until an adequate seal or disappearance of the thrombus was confirmed by TEE imaging.

**Table 3:** Antithrombotic treatment regimen within 45 days post-LAAC.

**Variables**	**Overall cohort**	**Score 0–2**	**Score 3–4**	**Score** ≥**5**	***P*** **-value for trend**
	***n*** = **401**	***n*** = **75**	***n*** = **188**	***n*** = **138**	
Duration of hospital stay post LAAC					
Warfarin, *n* (%)	2 (0.5)	1 (1.3)	1 (0.5)	0 (0)	0.191
Aspirin plus warfarin, *n* (%)	49 (12.2)	9 (12.0)	24 (12.8)	16 (11.6)	0.881
Aspirin plus LMWH, *n* (%)	236 (58.9)	50 (66.7)	120 (63.8)	66 (47.8)	0.003
Aspirin plus DOACs, *n* (%)	62 (15.5)	6 (8.0)	23 (12.2)	33 (23.9)	<0.001
Aspirin plus clopidogrel, *n* (%)	48 (12.0)	9 (12.0)	19 (10.1)	20 (14.5)	0.455
None, *n* (%)	4 (1.0)	1 (1.3)	0 (0)	3 (2.2)	0.333
Period from discharge to 45 days post LAAC					
OAC, *n* (%)	232 (57.9)	47 (62.7)	115 (61.2)	70 (50.7)	0.056
Aspirin plus OAC, *n* (%)	114 (28.4)	21 (28.0)	49 (26.1)	44 (31.9)	0.429
Aspirin plus clopidogrel, *n* (%)	51 (12.7)	8 (10.7)	23 (12.2)	20 (14.5)	0.401
None, *n* (%)	4 (1.0)	1 (1.3)	0 (0)	3 (2.2)	0.333

*Categorical variables are expressed as frequencies (n) and percentages (%). LAAC, left atrial appendage closure; LMWH, low-molecular-weight heparin; DOACs, direct oral anticoagulants; OAC, oral anticoagulant*.

### Long-term outcomes

All participants were followed up. The mean follow-up duration was 803 days (2.2 years), which corresponded to 882.2 patient-years in the overall cohort, with 176.1, 431.6, and 274.5 patient-years in the score 0–2, score 3–4, and score ≥5 groups, respectively. No significant differences were found in follow-up duration among the three groups (*P* = 0.115 for trend).

Table 4 shows the long-term outcomes after LAAC across the three score groups. In the overall cohort, the incidence of thromboemboli was 3.2%, 2.0% for ischemic stroke, and 1.3% for TIA. Major bleeding events occurred in 21 cases (5.2%), and the majority of major bleeding events (16 cases) were attributed to gastrointestinal bleeding. DRT was observed in 20 cases (5.0%) by TEE visit. Furthermore, a total of 59 deaths (14.7%) occurred in the overall cohort, among which cardiovascular and non-cardiovascular mortality rates were 6.7% (27 cases) and 8.0% (32 cases), respectively. However, the incidence rates of thromboembolism, major bleeding, DRT, all-cause mortality, cardiovascular mortality, non-cardiovascular mortality, and combined efficacy endpoints were comparable among the three score subgroups, respectively [each *P* = no significance (NS) for trend] (Table 4).

**Table 4:** Outcome events during follow-up.

**Variables**	**Overall cohort**	**Score 0–2**	**Score 3–4**	**Score** ≥**5**	***P*** **–value for trend**
	***n*** = **401**	***n*** = **75**	***n*** = **188**	***n*** = **138**	
Follow-up time, (days) (mean ± standard deviation)	803.3 ± 541.1	857.5 ± 528.0	838.2 ± 527.3	726.3 ± 561.7	0.115
Thromboembolism, *n* (%)	13(3.2)	2 (2.7)	7 (3.7)	4 (2.9)	0.987
Ischemic stroke, *n* (%)	8(2.0)	2(2.7)	3(1.6)	3(2.2)	0.897
TIA, *n* (%)	5(1.3)	1(1.3)	2(1.1)	2(1.5)	0.892
Systemic embolism, *n* (%)	0	0	0	0	—
Major bleeding, *n* (%)	21 (5.2)	3 (4.0)	10 (5.3)	8 (5.8)	0.592
Intracranial hemorrhage, *n* (%)	3(0.8)	0	2 (1.1)	1 (0.7)	0.185
GI bleeding, *n* (%)	16 (4.0)	3 (4.0)	7(3.7)	6 (4.4)	0.862
Other major bleeding, *n* (%)	2(0.5)	0	1(0.5)	1(0.7)	0.495
DRT, *n* (%)	20(5.0)	3(4.0)	8(4.3)	9(6.5)	0.357
All-cause mortality, *n* (%)	59 (14.7)	7 (9.3)	30 (16.0)	22 (15.9)	0.256
Cardiovascular mortality, *n* (%)	27(6.7)	4 (5.3)	13 (6.9)	10 (7.3)	0.623
Non-cardiovascular mortality, *n* (%)	32(8.0)	3 (4.0)	17 (9.0)	12 (8.7)	0.304
Combined efficacy endpoints, *n* (%)	70 (17.5)	9 (12.0)	35 (18.6)	26 (18.8)	0.267

*Continuous data are reported as means and standard deviation. Categorical variables are expressed as frequencies (n) and percentages (%). TEE, transesophageal echocardiography; TIA, transient ischemic attack; DRT, device-related thrombus; GI, gastrointestinal*.

**Figure 1:**
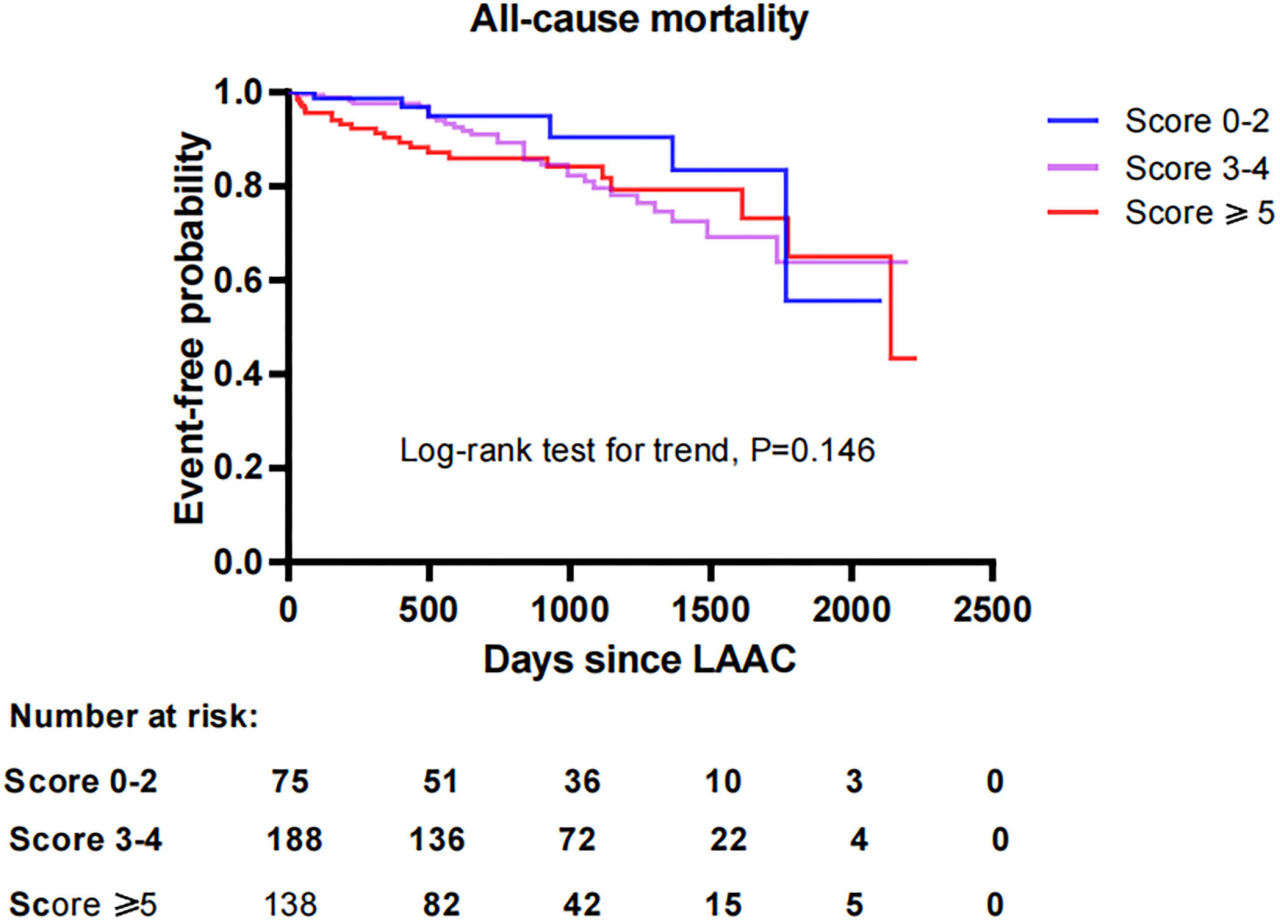
Comparison of the cumulative ratio of freedom from all-cause mortality in different subgroups. LAAC, left atrial appendage closure.

**Figure 2:**
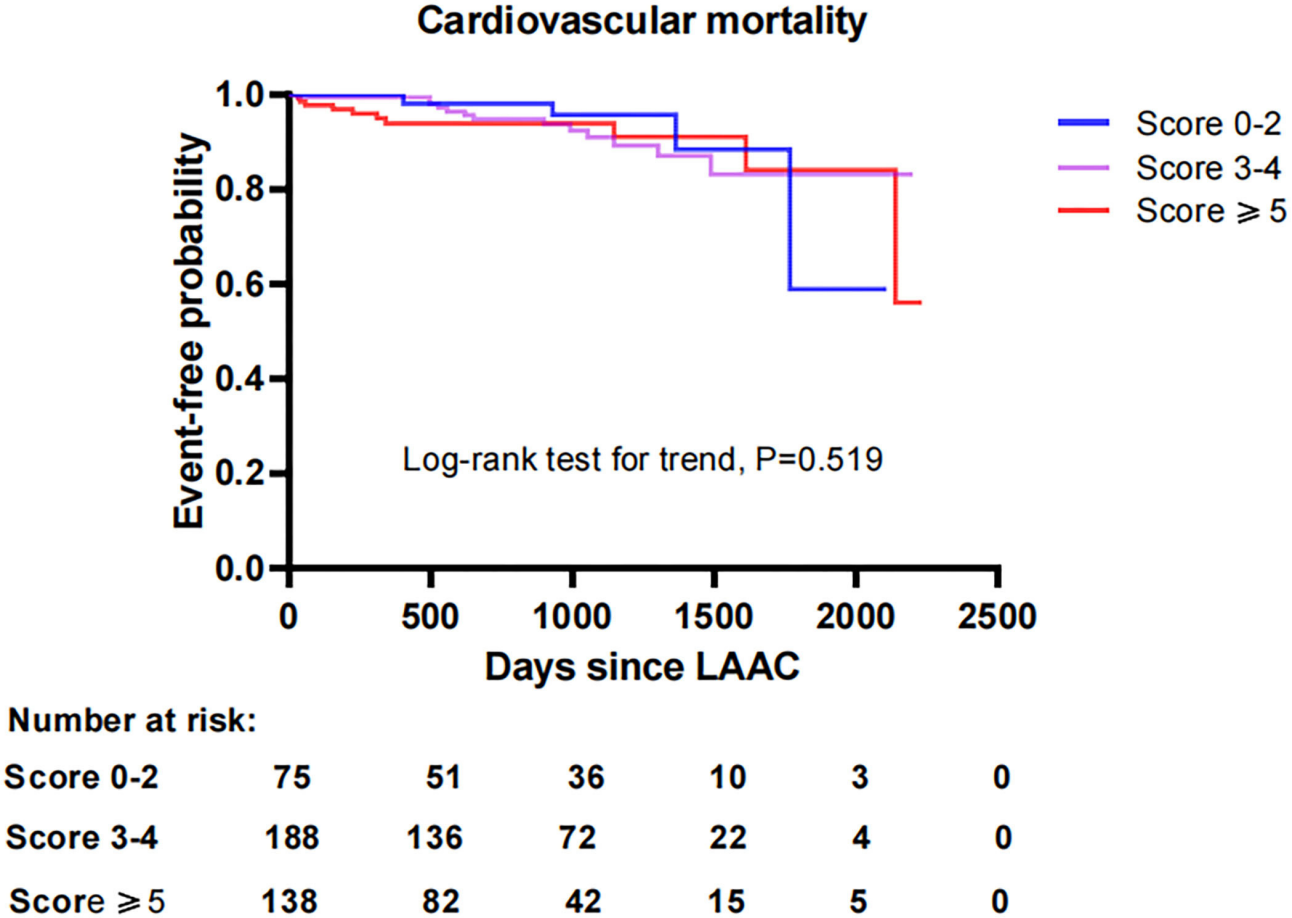
Comparison of the cumulative ratio of freedom from cardiovascular mortality in different subgroups. LAAC, left atrial appendage closure.

**Figure 3:**
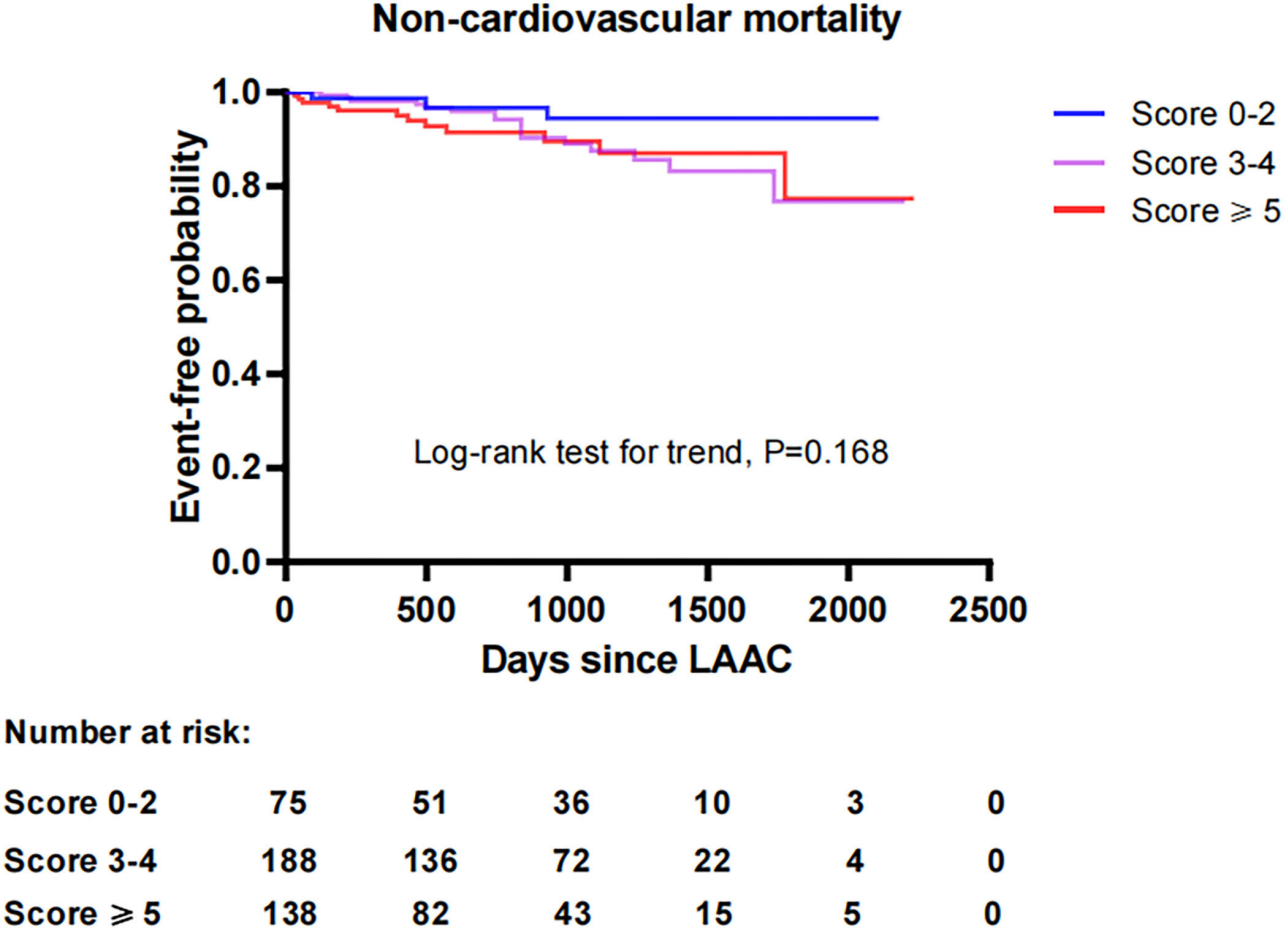
Comparison of the cumulative ratio of freedom from non-cardiovascular mortality in different subgroups. LAAC, left atrial appendage closure.

To investigate the impact of CHA_2_DS_2_-VASc score on survival after LAAC, we performed a Kaplan-Meier survival curve estimation, which showed that the cumulative ratio of freedom from all-cause mortality (log-rank test for trend, *P* = 0.146), cardiovascular mortality (log-rank test for trend, *P* = 0.519), or non-cardiovascular mortality (log-rank test for trend, *P* = 0.168) did not differ significantly by CHA_2_DS_2_-VASc score group, respectively (Figures 1–3, respectively).

### Effectiveness of LAAC on annual risk reduction of thromboembolism by CHA_2_DS_2_-VASc score

**Figure 4:**
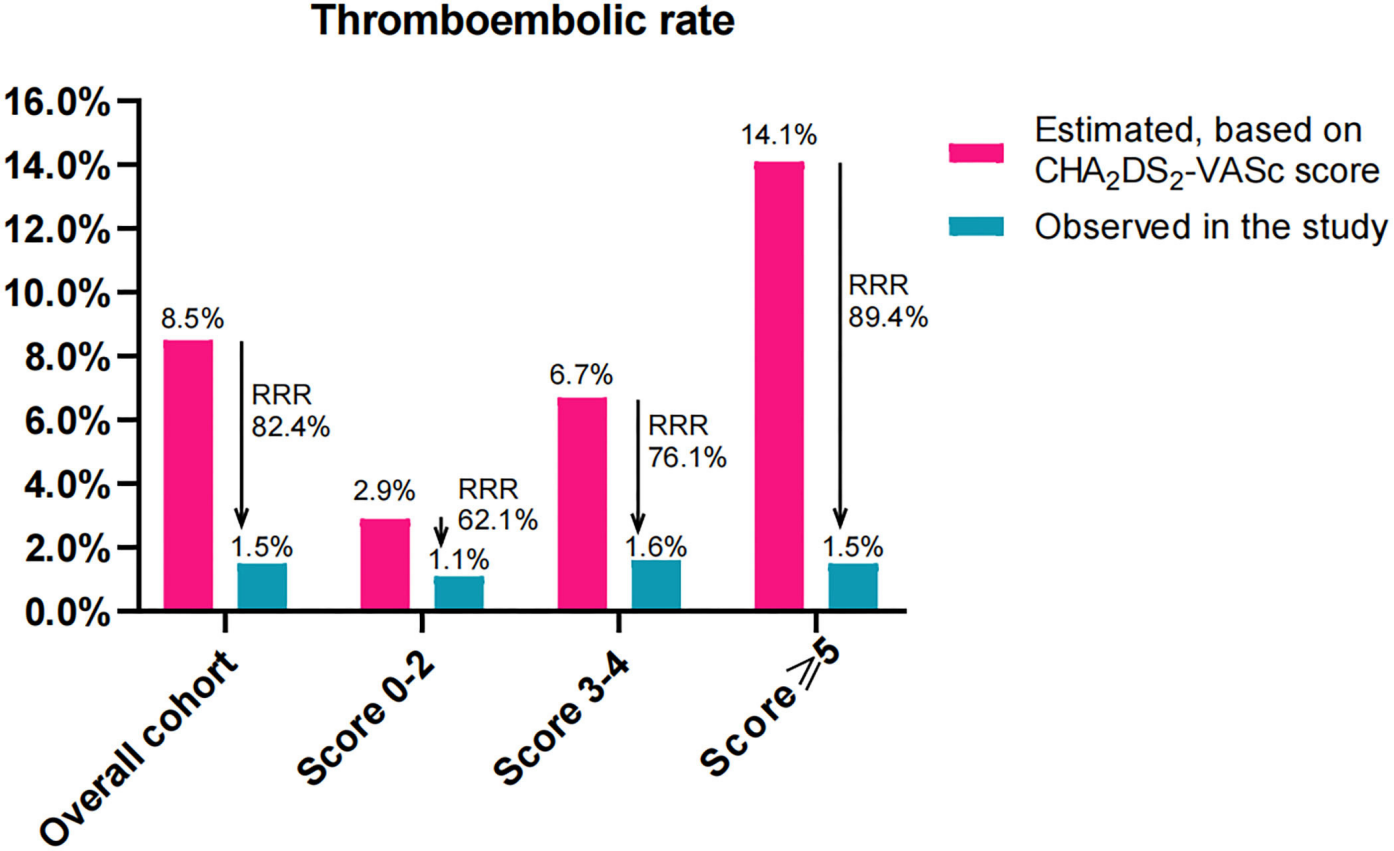
Effectiveness of LAAC in risk reduction of thromboembolism (100 patient-years) during the follow-up in the study groups divided according to CHA_2_DS_2_-VASc score. RRR, relative risk reduction.

The estimated annualized thromboembolic rates were 8.5%, 2.9%, 6.7%, and 14.1% in the overall cohort, score 0–2, score 3–4, and score ≥5 groups, respectively. However, the observed annualized thromboembolic rates were 1.5%, 1.1%, 1.6%, and 1.5%, which yielded a RRR of 82.4% (RR: 5.667, 95% CI: 2.475–13.06, *P* < 0.001), 62.1% (RR: 2.000, 95% CI: 0.267–15.10, *P* = 1.000), 76.1% (RR 4.333, 95% CI: 1.353–14.03, *P* = 0.019), and 89.4% (RR 10.0, 95% CI: 2.675–38.13, *P* < 0.001), respectively (Figure 4). Accordingly, the NNT to prevent one thromboembolic event for LAAC was 14 (95% CI: 10–26), 75, 19 (95% CI: 10–127), and 8 (95% CI: 5–16), respectively.

For the comparison of RRR among the three subgroups, the level of RRR increased with CHA_2_DS_2_-VASc score (*P* < 0.001 for trend). Compared with patients in the score 0–2 group, the level of RRR was significantly increased in those with score 3–4 (RR: 0.824, 95% CI: 0.666–0.981, *P* = 0.033) and score ≥5 (RR: 0.703, 95% CI: 0.572–0.829, *P* < 0.001) groups, respectively. Meanwhile, the level of RRR was also markedly greater in patients with score ≥5 than that in those with score 3–4 (RR: 0.853, 95% CI: 0.770–0.943, *P* = 0.002).

### Effectiveness of LAAC on annual risk reduction of major bleeding by CHA_2_DS_2_-VASc score

**Figure 5:**
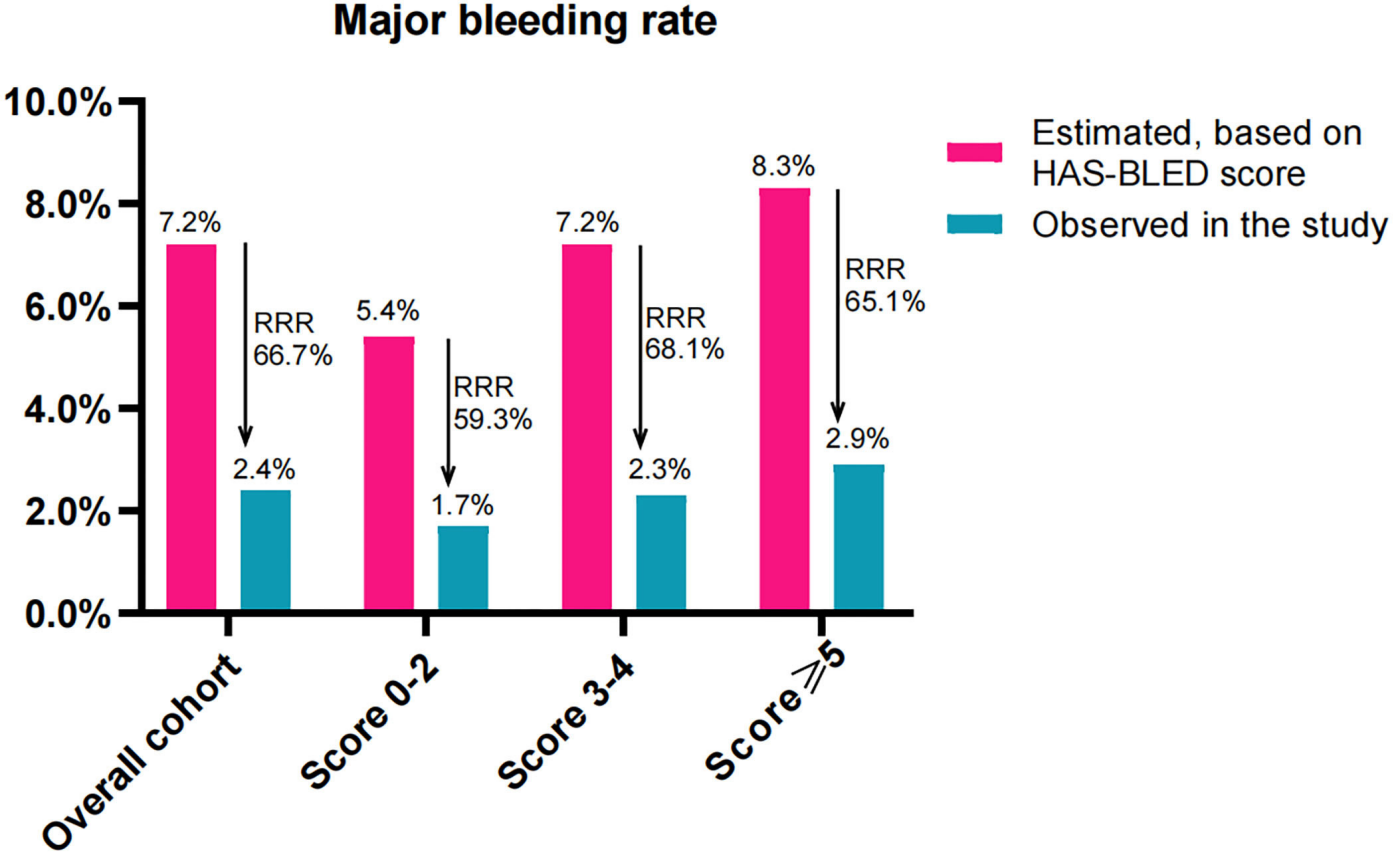
Effectiveness of LAAC in risk reduction of major bleeding (100 patient-years) during the follow-up in the study groups divided according to CHA_2_DS_2_-VASc score. RRR, relative risk reduction.

The estimated annualized major bleeding rates were 7.2%, 5.4%, 7.2%, and 8.3% in subjects of overall cohort, score 0–2, score 3–4, and score ≥5, respectively. However, the observed annualized major bleeding rates were 2.4%, 1.7%, 2.3%, and 2.9%, which constituted an RRR of 66.7% (RR: 3.048, 95% CI: 1.912–4.881, *P* < 0.001), 59.3% (RR: 3.333, 95% CI: 1.038–10.94, *P* = 0.078), 68.1% (RR 3.100, 95% CI: 1.594–6.09, *P* < 0.001), and 65.1% (RR 2.875, 95% CI: 1.366–6.128, *P* = 0.007), respectively (Figure 5). Accordingly, the NNT to prevent one major bleeding event for LAAC was 9 (95% CI: 7–6), 10, 9 (95% CI: 6–22), and 9 (95% CI: 5–35), respectively.

When comparing the RRR of major bleeding among the three subgroups, the extent of RRR did not differ significantly (*P* = 0.587 for trend). No statistically significant differences in the extent of RRR were found between patients score 3–4 and score 0–2 (RR: 0.881, 95% CI: 0.702–1.069, *P* = 0.250), and between patients score ≥5 and score 3–4 (RR: 1.044, 95% CI: 0.895–1.228, *P* = 0.634) or even score 0–2 (RR: 0.92, 95% CI: 0.726–1.136, *P* = 0.460).

## Discussion

This study demonstrated the following findings. (1) For AF patients, the higher the CHA_2_DS_2_-VASc score, the higher the comorbidity; however, no statistically significant differences were observed in implantation success or periprocedural complications along all score groups after LAAC. (2) The long-term outcomes and the cumulative ratio of freedom from mortality were comparable across all score groups. (3) LAAC decreased the thromboembolic risk in the overall cohort, score 3–4, and score ≥5 groups compared with estimated risk. The level of RRR of thromboembolism increased with CHA_2_DS_2_-VASc score among the three score subgroups. (4) LAAC lowered the major bleeding risk in the overall cohort, score 3–4, and score ≥5 groups compared with estimated risk, but the extent of RRR in major bleeding did not differ among subgroups.

The baseline clinical characteristics of the overall cohort were similar to those reported in previous randomized clinical trials, except for a higher proportion of patients ≥75 years of age and a slightly lower incidence of hypertension (6). We noted that the score ≥5 group not only had the oldest patients but also had the highest percentage of women and had a significantly increased prevalence of concomitant multimorbidity, such as hypertension, CHD, diabetes, CHF, previous stroke, previous major bleeding, and CKD, as well as the highest HAS-BLED scores. This finding is expected as many of the aforementioned clinical variables are scoring criteria for the CHA_2_DS_2_-VASc scale (13). In regard to the high HAS-BLED score correlating with a higher CHA_2_DS_2_-VASc score, scoring criteria for the HAS-BLED scale are similar to those of the CHA_2_DS_2_-VASc scale (14). Previous studies showed that the clinical risk of stroke or death in patients with AF increased with increasing CHA_2_DS_2_-VASc score (15, 16). Yoon et al. reported that accumulation of risk factors with increasing CHA_2_DS_2_-VASc score may translate to greater thromboembolism risk in AF patients at 10-year follow-up (17). Therefore, patients with higher CHA_2_DS_2_-VASc score were deemed as high-risk groups in our study. Nevertheless, the device implant success rate was consistent regardless of CHA_2_DS_2_-VASc score, and the incidence rate of periprocedural complications within 7 days did not demonstrate a significant increasing trend among patients with elevated CHA_2_DS_2_-VASc scores post-LAAC. Our implantation success rate was similar to that in the EWOLUTION registry, but the total incidence of periprocedural complications through 7 days was lower (18). Overall, the results showed that LAAC was equally safe in AF patients, regardless of CHA_2_DS_2_-VASc score.

With respect to the impact of CHA_2_DS_2_-VASc score on efficacy and long-term prognosis of LAAC, Ivănescu reported that major adverse cardiovascular and cerebrovascular events, including myocardial infarction, stroke, and mortality, increased with CHA_2_DS_2_-VASc score in AF patients (11). NVAF patients with increased CHA_2_DS_2_-VASc scores are not only considered prone to thromboembolic events but also associated with an increased risk of major bleeding in patients receiving oral warfarin or rivaroxaban (19). Furthermore, a small sample study suggested the possible role of CHA_2_DS_2_-VASc score in the development of DRT after LAAC (20). Even in patients with other conditions such as chronic kidney disease or CHD regardless of the presence of AF, CHA_2_DS_2_-VASc score was also identified to be significantly associated with worse outcomes (21, 22). However, did the correlation between increased CHA_2_DS_2_-VASc score and worse prognosis still present in patients with AF treated with LAAC? Our study showed that patients with higher CHA_2_DS_2_-VASc score did not experience worse outcomes in the incidences of thromboembolism, major bleeding, DRT, mortality risk, and combined efficacy endpoints in comparison to those with a lower score after LAAC during long-term follow-up. The higher incidences of adverse clinical outcomes attributed to higher CHA_2_DS_2_-VASc score were not observed in patients with higher scores undergoing LAAC in this study. Especially for the difference in mortality by CHA_2_DS_2_-VASc score, survival curves calculated by Kaplan-Meier estimation demonstrated that the cumulative ratio of freedom from all-cause mortality, cardiovascular mortality, or non-cardiovascular mortality was similar across the all score groups after LAAC, respectively. These results were not in accordance with those in other intervention studies that increases in CHA_2_DS_2_-VASc score were correlated with poor outcomes in AF patients or those receiving anticoagulation therapy (11, 19, 23). Previous studies also revealed that patients with AF, as well as those treated with oral anticoagulants who were at higher risk for major adverse cardiovascular and cerebrovascular events, could be identified by CHA_2_DS_2_-VASc score (11, 24). However, it was noteworthy that our study demonstrated that the CHA_2_DS_2_-VASc score did not significantly affect the risks of major adverse outcomes in AF patients who underwent LAAC during long-term follow-up. This might be due to the substantial differences in efficacy derived from different intervention strategies. LAAC vs. DOACs may have favorable effectiveness in reducing the risk of major bleeding and mortality among high-risk patients with AF (25). Furthermore, patients with NVAF who previously underwent LAAC had better neurological outcomes after acute ischemic stroke events in comparison with patients on warfarin (26). Therefore, these significant advantages of LAAC intervention over OAC therapy in high-risk AF patients may lead to the inconsistency regarding the influence of CHA_2_DS_2_-VASc score on outcomes among NVAF patients treated with LAAC and OAC. In fact, our findings suggest that LAAC may eliminate or normalize the difference of long-term adverse outcomes by CHA_2_DS_2_-VASc score in AF patients.

In the overall cohort, the observed yearly thromboembolic rate was as low as 1.5%, representing a significant RRR of 82.4% in thromboembolism after LAAC compared with the expected risk by CHA_2_DS_2_-VASc score, which was in line with findings in LAAC registries with a risk reduction of 84% in the composite endpoint of ischemic stroke/TIA/systemic embolism (27). Despite the higher annual rate of expected thromboembolism due to an increase in CHA_2_DS_2_-VASc score in AF patients, subgroup analysis showed that the observed annual thromboembolic rates did not significantly increase with CHA_2_DS_2_-VASc score point. Conversely, the RRR in thromboembolism increased significantly with CHA_2_DS_2_-VASc score across all score groups with an additional significant RRR in score 3–4 and ≥5 groups. In fact, patients with the highest CHA_2_DS_2_-VASc scores experienced the greatest RRR in thromboembolism post-LAAC. To gain a better understanding of the clinical relevance to this thromboembolism reduction, the NNTs for LAAC intervention were calculated. The NNT for LAAC intervention in the score ≥5 group was particularly low-−8 (95% CI: 5–16) over a mean 2.2 years of follow-up—which was notable in comparison with the NNTs in the 0–2 and 3–4 groups of 75 and 19, respectively. These results demonstrated that the higher the CHA_2_DS_2_-VASc score, the greater the benefit of LAAC intervention in risk reduction of thromboembolism in AF patients. Our conclusions were different from other reports, in which AF patients with higher CHA_2_DS_2_-VASc score were still at higher risk and did not benefit more from optimal anticoagulation or ablation in risk reduction of the thromboembolic event or other adverse outcomes (28, 29). So, our findings highlighted the greater efficacy of LAAC intervention in the RRR of thromboembolism in higher score groups but especially in patients with the highest CHA_2_DS_2_-VASc score. This may be one of the reasons why no worse outcomes were found in patients with vs. without a higher CHA_2_DS_2_-VASc score after LAAC. Further study with large sample size is needed to verify the conclusions in such a special group.

Several studies reported that CHA_2_DS_2_-VASc score is also associated with the risk of major bleeding in patients who underwent anticoagulation therapy (19, 30). Therefore, the expected annualized major bleeding rates based on HAS-BLED score were evidently higher in patients with higher CHA_2_DS_2_-VASc scores in our study. However, the observed annual major bleeding rate after LAAC was statistically lower than the expected risk in the overall cohort group with a RRR of 66.7% in major bleeding, which was similar to the risk reduction of 70% of major bleeding in AF patients treated with combined LAAC and catheter ablation (27). Recent randomized control trials confirmed that LAAC could yield a favorable outcome in major bleeding compared with treatment by warfarin or DOACs in AF patients (6, 31). Our subgroup analysis also indicated that patients with higher CHA_2_DS_2_-VASc score rather than those with the score 0–2 exhibited lower observed annual major bleeding rates compared with the HAS-BLED predicted rates. Interestingly, unlike RRR in thromboembolism, the level of RRR in major bleeding did not increase statistically with CHA_2_DS_2_-VASc score across the three groups. This suggests that LAAC may offer major bleeding benefits in high-risk patients and that the efficacy of LAAC on the extent of RRR in major bleeding does not appear to be influenced by CHA_2_DS_2_-VASc score.

There are several limitations in this study. First, this is a non-randomized observational study that may exist a possible selection bias and have a decreased power in assessing LAAC efficacy and long-term outcomes by CHA_2_DS_2_-VASc score. Second, we investigated the effect of LAAC on thromboembolism and major bleeding events using comparisons with historically expected risks instead of risks of control groups, which limits the scope of our conclusions as risk scores do not contain all individual patient factors. Finally, the relatively small number of patients may not be enough to evaluate the LAAC effectiveness by CHA_2_DS_2_-VASc score.

In summary, despite NVAF patients with higher CHA_2_DS_2_-VASc score being susceptible to an increased risk of major adverse cardio-cerebrovascular events, they did not exhibit lower implantation success, more periprocedural complications, or worse long-term outcomes after LAAC compared to those with lower CHA_2_DS_2_-VASc score. LAAC resulted in significant decreases in the risks of thromboembolism and major bleeding over the expected risks in patients with higher scores, and the level of RRR in thromboembolism rather than in major bleeding increased with CHA_2_DS_2_-VASc score among the three score groups. This study suggests that LAAC may eliminate the difference in long-term adverse outcomes by CHA_2_DS_2_-VASc score, which may be partly attributed to more benefits provided by LAAC intervention in AF patients with a higher CHA_2_DS_2_-VASc score versus those with a lower score.

## Data Availability Statement

The original contributions presented in the study are included in the article/supplementary material, further inquiries can be directed to the corresponding author.

## Ethics Statement

The studies involving human participants were reviewed and approved by the Ethics Committee of Helmut-G.-Walther Klinikum, Lichtenfels, Germany and Zhengzhou Ninth People's Hospital, Zhengzhou, China. The patients/participants provided their written informed consent to participate in this study.

## Author contributions

MiZ designed the study, performed the data collection and data analysis, interpreted the patient data, and wrote the manuscript. MeZ performed data collection, statistical analysis of data, production of the statistical graph, and revision of the manuscript. CH, FP, and NH performed a critical revision of the manuscript. JW, QY, and ZM contributed to the data analysis and discussion of the results. JY contributed to the study design, interpretation of data, and critical revision of the manuscript. All authors read and approved the final version of the manuscript.

## Conflict of interest

JY is a consultant to Boston Scientific and LifeTech Scientific. The remaining authors declare that the research was conducted in the absence of any commercial or financial relationships that could be construed as a potential conflict of interest.

## Publisher's note

All claims expressed in this article are solely those of the authors and do not necessarily represent those of their affiliated organizations, or those of the publisher, the editors and the reviewers. Any product that may be evaluated in this article, or claim that may be made by its manufacturer, is not guaranteed or endorsed by the publisher.
